# Colo-cutaneous fistula in the setting of complicated sigmoid diverticulitis previously managed with percutaneous drainage: a case report

**DOI:** 10.1093/jscr/rjae674

**Published:** 2024-12-26

**Authors:** Natalia Correa

**Affiliations:** DeWitt Daughtry Family Department of Surgery, University of Miami Miller School of Medicine, 1611 NW 12th Ave, Miami, FL 33136, United States; University of Miami Miller School of Medicine, 1306 Stanford Drive, Suite 1210, Coral Gables, FL 33146, United States; University of Miami Holy Cross Hospital General Surgery Residency Program, 1900 E Commercial Blvd, Ste 201, Fort Lauderdale, FL 33308, United States

**Keywords:** percutaneous drainage, fistula, colorectal, diverticulitis, abscess, diverticular disease, colon

## Abstract

Colo-cutaneous fistulas are a rare complication of diverticular disease. Percutaneous drainage offers a promising alternative to surgical intervention in the management of complicated diverticular disease with abscess formation. Recent case studies and literature reviews support its efficacy in achieving abscess resolution and reducing the need for surgery. However, careful patient selection, technical proficiency, and multidisciplinary management are critical to optimizing outcomes. As the body of evidence grows, percutaneous drainage is likely to play an increasingly important role in the therapeutic arsenal against complicated diverticular disease; however, the risk of complications, particularly fistula formation, must be carefully considered. Our case presents a rare incident of a colo-cutaneous fistula occurring after CT-guided percutaneous drainage of a pericolic abscess secondary to perforated sigmoid diverticulitis that failed medical management. The patient ultimately underwent Hartmann’s procedure where the diseased sigmoid colon, adhered small bowel, and fistula tract were excised with an end colostomy creation.

## Introduction

Colonic diverticular disease is a common and significant issue in Western societies, particularly among the aging population. The management of this condition, especially when complicated by abscess formation, involves a multidisciplinary approach including medical, surgical, and interventional specialists. While traditional surgical intervention has been the mainstay of treatment for complicated diverticular disease, recent evidence highlights percutaneous drainage as an effective and less invasive alternative. Percutaneous drainage has shown high success rates and reduced morbidity, but it is not without risks such as fistula formation. As research advances, percutaneous drainage is likely to become an increasingly important tool in managing complicated diverticular disease.

## Case report

**Figure 1:**
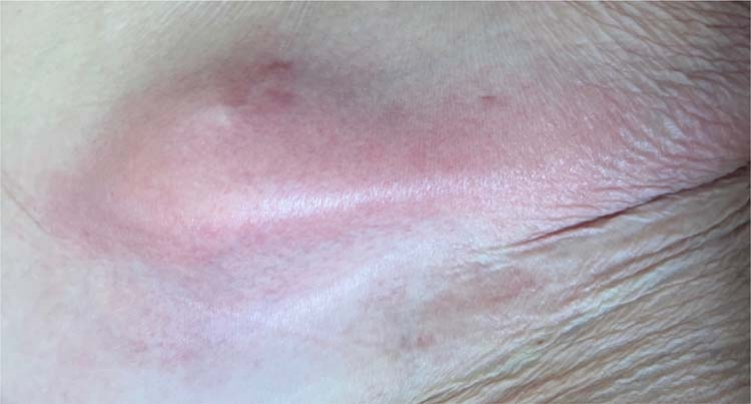
Anterior abdominal wall abscess from colo-cutaneous fistula.

**Figure 2:**
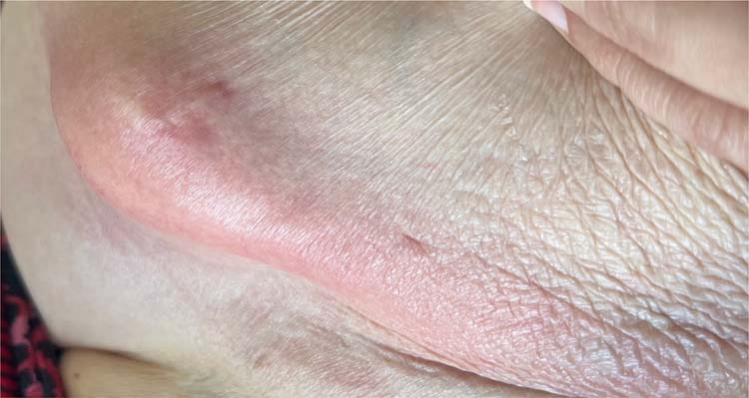
Anterior abdominal wall abscess from colo-cutaneous fistula.

**Figure 3:**
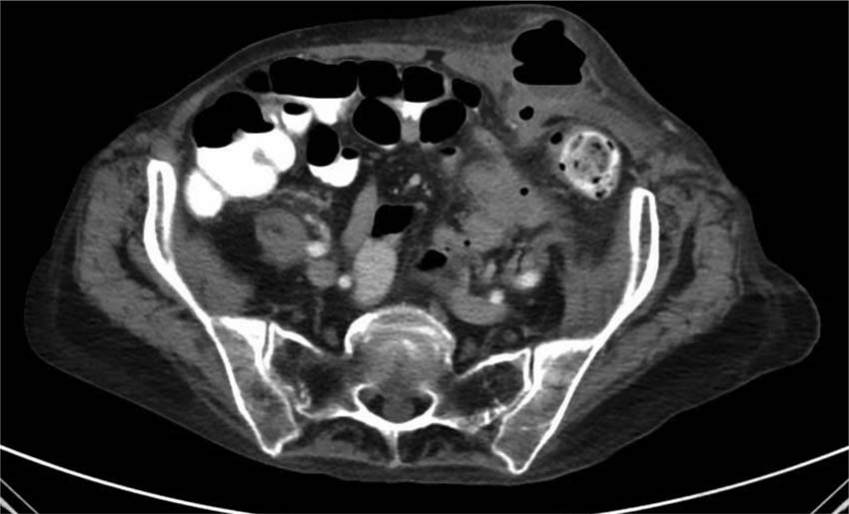
CT from readmission with anterior abdominal wall abscess and left pelvic abscess.

A 65-year-old female with a history of hyperlipidemia, hypertension, hip replacement, lumbar spinal fusion, chronic pain management, and prior laparotomy for endometriosis presented with a 3-day history of nausea, vomiting, and abdominal pain. She had no prior history of diverticulosis or colonoscopy. On examination, she was afebrile and hemodynamically stable. Physical examination revealed left lower quadrant tenderness, swelling, erythema, and fluctuance, suggesting an abdominal wall abscess (Figs 1 and 2). Laboratory tests showed leukocytosis with a count of 24.6 and a positive urinalysis with 2+ leukocytes. A CT scan of the abdomen and pelvis revealed a 3.8 × 3.6 cm air and fluid pocket in the low left anterior abdominal wall, indicative of an abscess likely at the site of a previous drain (Fig. 3). Additionally, there was a 2.8 × 1.8 cm irregular air pocket in the left pelvis, where an abscess had been identified in prior imaging.

**Figure 4:**
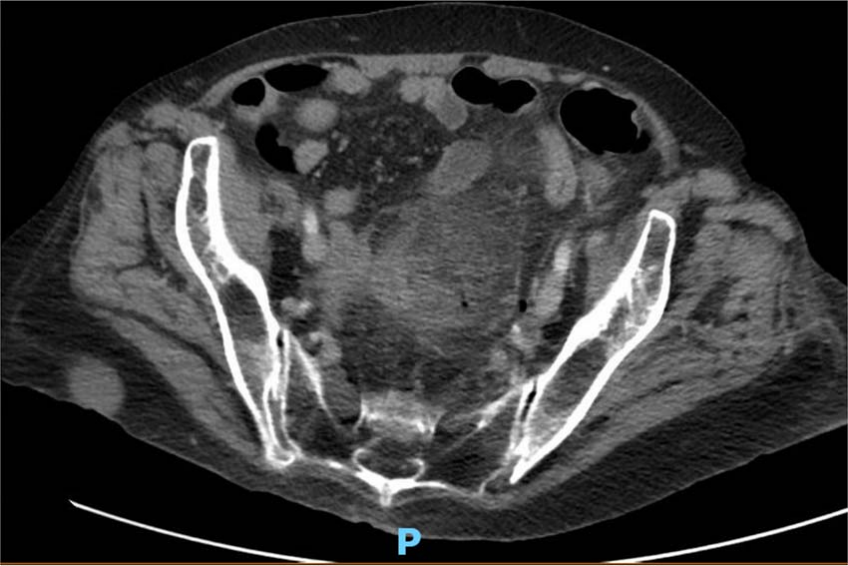
CT with perforation from prior admission.

**Figure 5:**
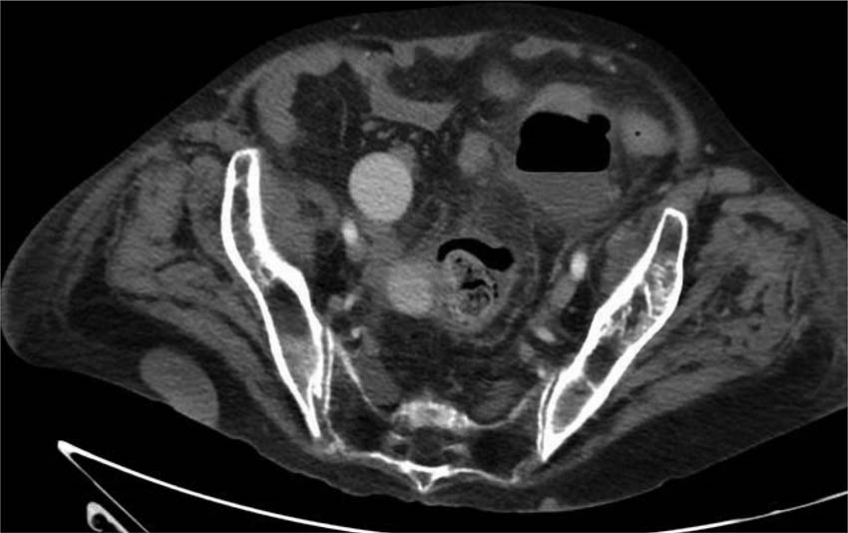
CT with abscess developed after initial antibiotic treatment.

**Figure 6:**
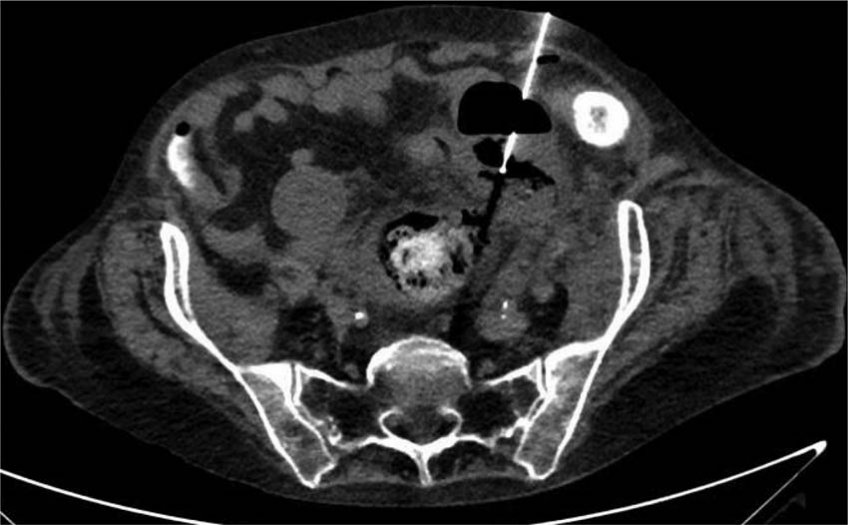
CT-guided percutaneous drainage of abscess.

**Figure 7:**
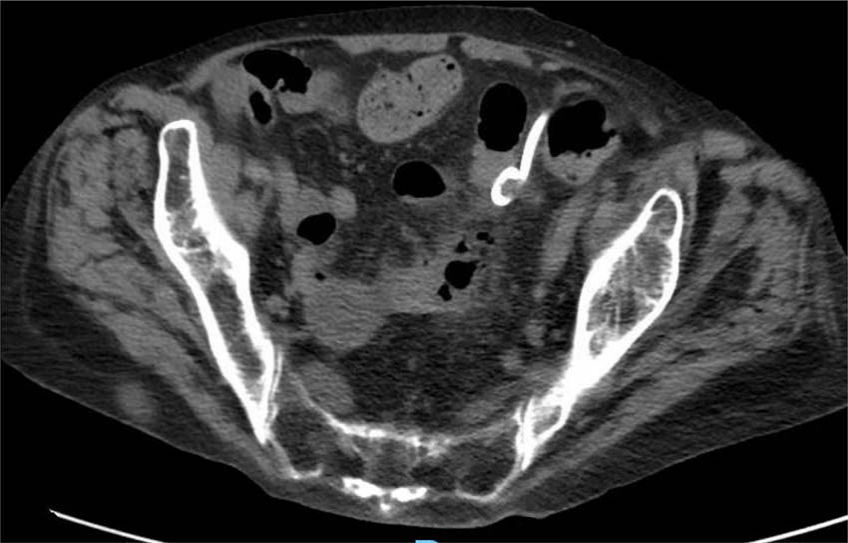
CT showing resolution of abscess with IR pigtail drain in place.

One month prior, the patient was admitted for complicated perforated sigmoid diverticulitis. Treatment included piperacillin-tazobactam (Zosyn), bowel rest, and fluid resuscitation. On hospital Day 6, her condition worsened with increased nausea, vomiting, and an elevated WBC count of 15. CT imaging revealed a 4.8 × 4.4 × 4.0 cm diverticular abscess, leading to CT-guided percutaneous drainage by interventional radiology (Figs 4–7). Cultures grew *Citrobacter sedlakii* and *Enterococcus faecium* vancomycin resistant Enteroccus (VRE), and treatment was adjusted to include Bactrim, followed by daptomycin and levofloxacin. The abscess resolved after 3 weeks, and the drain was removed before discharge. The patient was instructed to follow up with her surgeon and undergo a 6-week interval colonoscopy.

**Figure 8:**
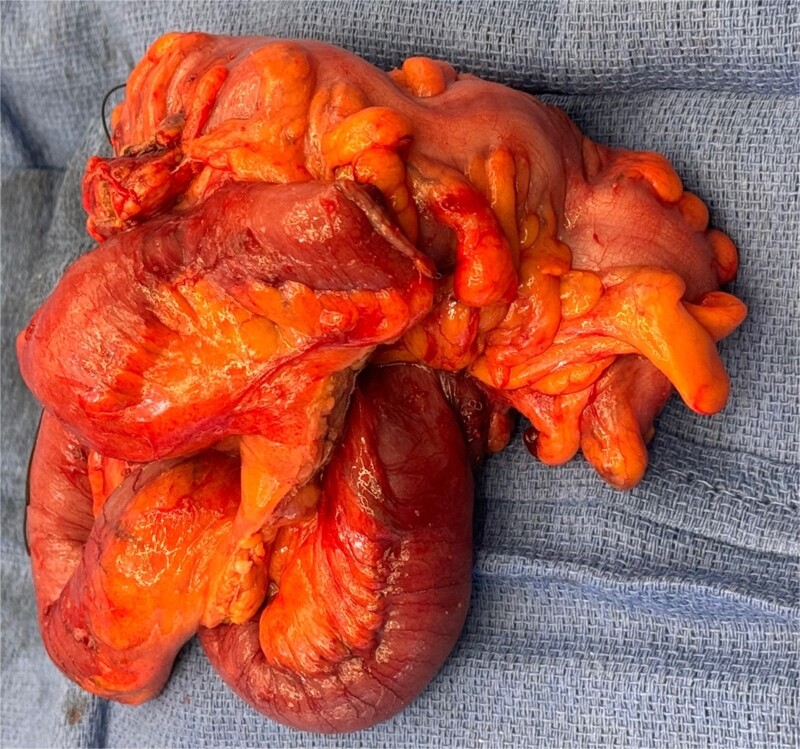
Intraoperative findings of colo-cutaneous fistula, sigmoid colon, and adhered small bowel.

Upon readmission, the patient underwent a bedside incision and drainage (I&D) procedure, which yielded air but no pus or feculent material. Probing revealed a connection to the abdominal cavity, with cultures positive for heavy *E. coli*. The wound was packed with iodoform, which produced copious purulent material the following day. Patient was subsequently taken to the operating room for exploratory laparotomy, sigmoidectomy, small bowel resection with primary anastomosis, and colostomy creation (Hartmann’s procedure). Intraoperative findings were concerning for colo-cutaneous fistula with a firm mass in the center of sigmoid colon where multiple loops of small bowel were adhered (Fig. 8). Pathology indicated marked acute and chronic diverticulitis with abscess formation, transmural inflammation, fibrosis, and extensive adhesions. Postoperatively, the patient was managed with nasogastric tube decompression, bowel rest, and dilaudid patient controlled analgesia pump.

## Discussion

Recent studies underscore the effectiveness of percutaneous drainage for managing diverticular abscesses. Garfinkle *et al.* [1] supported nonoperative management as a safe long-term solution, especially in the acute setting. Aquina *et al*. [2] found that for the average risk patient who underwent successful drainage of a diverticular abscess, observation without elective colectomy appeared to be a safe and reasonable option as 75% of patients will never suffer a recurrent episode of diverticulitis. Gaertner *et al*. [3] found that patients who underwent percutaneous drainage without subsequent colectomy had a recurrence-free survival at 7.4 years of 58%. Recurrence was significantly associated with an abscess larger than 5 cm, and all recurrences were managed nonoperatively. Additionally, Elagli *et al.* [4] had a study indicating percutaneous drainage of diverticular abscess was generally safe and effective, consistently with the reported success rates ranging between 74% and 93%.

Comparative studies reveal that percutaneous drainage generally has lower initial morbidity and shorter hospital stays compared with surgical intervention. Singh *et al*. [5] conducted a retrospective study that found percutaneous drainage can be used as a bridge before definitive surgery but also as a treatment option in high-risk surgical patients. It can reduce the need for major surgery and reduce the risk of a permanent stoma. Bachelani *et al*. [6] found that small abscesses showed a higher success rate, longer time to recurrence, and required surgery less often than large abscesses. A larger abscess size was associated with a higher risk of surgery and decreased time to failure.

Current guidelines recommend percutaneous drainage for abscesses >3 cm in hemodynamically stable patients without generalized peritonitis [7]. Smaller abscesses or microperforations may be managed conservatively with antibiotics alone. Advances in imaging, including CT and ultrasound, have improved the precision of percutaneous drainage, enhancing success rates. Effective percutaneous drainage depends on proper patient selection and the technical aspects of the procedure. Multidisciplinary collaboration among radiologists, surgeons, and gastroenterologists is crucial to optimize patient outcomes.

Despite its advantages, percutaneous drainage is associated with potential complications, including fistula formation. Gregersen *et al*. [8] noted that of patients undergoing percutaneous drainage, 2.5% experienced procedure-related complications, primarily enterocutaneous fistulas and small bowel lesions, and 15.5% needed adjustment or replacement of the drain. Fistulas may result from persistent inflammation or infection creating abnormal connections between the abscess cavity and adjacent organs. Risk factors for fistula formation include larger or more complex abscesses, comorbid conditions like diabetes or chronic kidney disease, and inadequate drainage. Management of fistulas involve a multidisciplinary approach. Small, asymptomatic fistulas may close spontaneously with conservative management, including bowel rest, total parenteral nutrition, and antibiotics. Persistent or large fistulas often require surgical intervention, which may involve resection of the affected bowel segment, primary repair, or creation of a stoma to divert fecal flow and facilitate healing.

Percutaneous drainage is an effective and less invasive treatment for diverticular abscesses, offering significant benefits over traditional surgical approaches, including reduced initial morbidity and shorter hospital stays. The growing body of evidence supports its use as a first-line treatment for stable patients, with surgical intervention reserved for those who do not respond to initial drainage. However, careful patient selection and management of potential complications are essential for optimizing outcomes. As evidence continues to support its efficacy and safety, percutaneous drainage is poised to play an increasingly important role in the management of complicated diverticular disease.
